# Cholecalciferol and muscle strength in hemodialysis patients: results from the randomized VITADIAL trial

**DOI:** 10.1093/ckj/sfag166

**Published:** 2026-05-21

**Authors:** Stanislas Bataille, Guillaume Seret, Frederic Lavainne, Philippe Giaime, Marianne Serveaux, Thomas Robert, Mickael Bobot, Laurence Vrigneaud, Guillaume Jean, Louis De Laforcade, Mathilde Prezelin-Reydit, Philippe Chauveau, Myriam Isnard, Laurent Samson, Angélique Colin, Frederique Boury, Axel Viton, Magali Claeys-Bruno, Nathalie Pedinielli

**Affiliations:** Phocean Institute of Nephrology, Nephrology Department, 13006, Marseille, France; ELSAN, Clinique Bouchard, Nephrology Department, Marseille, France; Association ECHO, Pôle Santé Sud, Le Mans, France; Association ECHO, Polyclinique de l’Atlantique, 44162 Saint Herblain, France; Phocean Institute of Nephrology, Nephrology Department, 13006, Marseille, France; ELSAN, Clinique Bouchard, Nephrology Department, Marseille, France; ATUP, Centre d'Hémodialyse, Marseille, France; Phocean Institute of Nephrology, Nephrology Department, 13006, Marseille, France; ELSAN, Clinique Bouchard, Nephrology Department, Marseille, France; ATUP, Centre d'Hémodialyse, Marseille, France; Département de Néphrologie et néphrogénomique, Hôpital Saint Joseph, Marseille, France; Centre de néphrologie et transplantation rénale APHM, Marseille, France; Aix Marseille Univ, INSERM 1263, INRAE 1260, Marseille, France; Centre de néphrologie, Hôpital privé La Louvière, Ramsay Santé, Lille,France; Centre d'Hémodialyse, NephroCare Tassin-Charcot, Ste Foy-Lès-Lyon, France; Service de Néphrologie, Centre hospitalier de Libourne, Libourne, France; Maison du REIN AURAD Aquitaine, Gradignan, France; Maison du REIN AURAD Aquitaine, Gradignan, France; ATIR, Centre d'Hémodialyse, Avignon, France; Centre de néphrologie et transplantation rénale APHM, Marseille, France; Association ECHO, Pôle Santé Sud, Le Mans, France; Association ECHO, Polyclinique de l’Atlantique, 44162 Saint Herblain, France; Service de Néphrologie, Centre hospitalier de Libourne, Libourne, France; Aix Marseille Univ, CNRS, IRD, IMBE, Marseille, France; Aix Marseille Univ, CNRS, IRD, IMBE, Marseille, France; Phocean Institute of Nephrology, Nephrology Department, 13006, Marseille, France

**Keywords:** cholecalciferol, chronic hemodialysis, chronic kidney disease–mineral and bone disorders (CKD MBD), muscle strength, vitamin D

## Abstract

**Background:**

Many factors contribute to muscle impairment during chronic kidney disease, among which vitamin D deficiency has been implicated. The aim of this study was to analyze whether cholecalciferol supplementation in hemodialysis patients with low 25-hydroxyvitamin vitamin D (25OHD) concentrations could improve handgrip strength (HGS).

**Methods:**

VITADIAL (Does Correction of 25 OH-VITAmin D With Cholecalciferol Supplementation Increase Muscle Strength in HemoDIALysis Patients?) is a prospective open-labeled French multicenter study in which chronic hemodialysis patients with a 25OHD ≤50 nmol/L were randomized to receive monthly oral 100 000 IU cholecalciferol or no vitamin D treatment during 6 months to assess whether cholecalciferol supplementation in vitamin D–deficient hemodialysis patients improves muscle strength as measured with HGS. The main objective of the study was to analyze whether a 6-month period of oral cholecalciferol improved the muscle strength of hemodialysis patients with low 25OHD levels.

**Results:**

Within the 270 hemodialysis patients from 10 hemodialysis centers included in the study, 143 patients with 25OHD ≤50 nmol/L were randomized: 70 were allocated to the cholecalciferol group and 73 to the control group. The two groups were comparable at inclusion. The cholecalciferol supplemented group's 25OHD rose to 89.78 ± 33.43 nmol/L at 6 months and remained ≤50 nmol/L for most patients in the control group, but HGS did not change significantly between randomization and end of the study in both groups (0.07 ± 3.40 kg and –0.19 ± 3.35 kg, respectively, *P*-value .682). No difference was found in terms of either autonomy or frailty.

**Conclusions:**

This highly powered study did not find any effect of cholecalciferol supplementation in muscle strength of hemodialysis patients with low 25OHD.

Trial registration: ClinicalTrials, NCT04262934. Registered 10 February 2020. Retrospectively registered, https://clinicaltrials.gov/ct2/show/NCT04262934

## Graphical Abstract

**Graphical Abstract:**
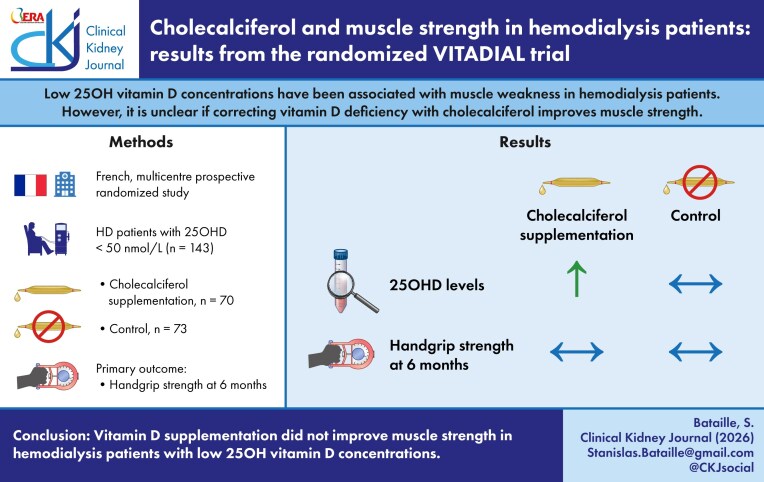

KEY LEARNING POINTS **What was known:**

- Low 25-hydroxyvitamin D (25OHD) levels have been associated to reduced muscle strength in observational studies in hemodialysis patients, suggesting a possible causal link.
- Previous randomized trials of cholecalciferol in dialysis patients were small, short or underpowered, and therefore unable to provide a definitive answer.
- Whether correcting vitamin D deficiency improves muscle strength or functional outcomes in hemodialysis patients remained unknown.

**This study adds:**

- In this adequately powered multicenter randomized trial, monthly cholecalciferol significantly increased serum 25OHD but did not improve handgrip strength.
- These results provide the strongest evidence to date that cholecalciferol supplementation does not enhance muscle strength in hemodialysis patients with low 25OHD levels.
- No benefit was observed on autonomy, frailty or functional performance despite biochemical correction of vitamin D deficiency.

**Potential impact:**

- Vitamin D supplementation should not be used with the expectation of improving muscle strength or functional capacity in hemodialysis patients.
- These findings support a more targeted and evidence-based use of cholecalciferol in routine dialysis care.

## INTRODUCTION

Muscle strength decreases as kidney failure progresses [1]. Low muscle strength affects more than 50% of hemodialysis patients and leads to impairment of activities of daily living [2–4]. Many factors contribute to muscle impairment during chronic kidney disease: some are directly linked to kidney failure such as uremic toxins accumulation, notably indoxyl sulfate [5–9], others are due to associated comorbidities such as malnutrition, chronic inflammation or lack of exercise.

Much *in vitro* data favors a role of vitamin D on muscle cells: vitamin D receptor is expressed by muscle cells [10]; 25-hydroxyvitamin D (25OHD) is implicated in myoblast cell proliferation in culture; vitamin D is implicated in the recruitment, proliferation and differentiation of satellite cells into mature muscle fibers in rats, and is essential for wounding repair [11]; and vitamin D is also required for mature muscle fiber contraction and mitochondrial metabolism [12].

In the general population and in chronic kidney disease (CKD) patients, numerous studies report that vitamin D insufficiency assessed by low 25OHD was associated with low muscle strength [13–18]. Our team has reported an independent “dose–effect” relationship between 25OHD and muscle strength measured by handgrip strength (HGS) in hemodialysis patients, favoring a normal threshold of 75 nmol/L for 25OHD in this population [13]. The VITADIAL study has emerged from these observations.

Nevertheless, association is not causation and the direct role of vitamin D supplementation on muscle strength has not been demonstrated to date: several studies of different designs failed to show a beneficial effect of supplementation on muscle strength in general population [19–21], or showed only a small effect [22]. Moreover, studies using high doses of cholecalciferol showed an increased risk of falls [23, 24].

Few studies analyzed the effect of vitamin D supplementation on muscle strength in hemodialysis patients. Marckman *et al*. randomized 52 CKD patients including 27 hemodialysis patients with 25OHD ≤50 nmol/L to an 8-week long treatment of 40 000 IU cholecalciferol per week or placebo. Cholecalciferol had no effect on muscle function (secondary objective) in this short and small study [25]. In the second study, Hewitt *et al*. included 60 hemodialysis patients with 25OHD <60 nmol/L to take 50 000 IU cholecalciferol or placebo, once per week for 8 weeks and then once per month for 4 months. This study failed to show any difference in muscle strength of upper and lower limb or functional performances between supplemented and non-supplemented patients, but patients were not severely deficient in vitamin D [26]. Singer *et al*. randomized 68 dialysis patients with 25OHD <50 nmol/L to 50 000 U/week oral cholecalciferol or matching placebo. The authors found no effect of cholecalciferol on HGS at 12 months, but unfortunately, the target sample size of 88 patients was not reached [27].

Our aim was therefore to build up a randomized study with enough power to analyze whether cholecalciferol supplementation in hemodialysis patients with low 25OHD concentrations could improve HGS.

## MATERIALS AND METHODS

Detailed study protocol and methods have already been published [28]. Briefly, VITADIAL is a prospective open-labeled French multicenter study in which hemodialysis patients with a 25OHD ≤50 nmol/L were randomized to receive either monthly oral 100 000 IU cholecalciferol or no vitamin D treatment for 6 months in order to assess whether cholecalciferol supplementation in vitamin D–deficient hemodialysis patients improves muscle strength.

### Population

Hemodialysis patients were recruited in 10 hemodialysis centers in France. Inclusion criteria were: on hemodialysis for >3 months, aged >18 years old. Non-inclusion criteria were: non-fluent French speaker, inability to answer questionnaires, pregnancy or breastfeeding, cognitive impairment, bedridden or predicted life expectancy <1 year, active cancer, uncontrolled hyperparathyroidism as defined by the KDIGO criteria [intact parathyroid hormone (iPTH) >9× normal laboratory maximal value], cinacalcet treatment, hypocalcemia <2.0 mmol/L, hypercalcemia >2.7 mmol/L, past osteoporosis fracture, treatment with active vitamin D, unable to perform handgrip measurement, cholecalciferol intolerance or allergy. All patients provided written consent and were informed of their right to withdraw from the study at any time.

### Study protocol

Patients with 25OHD ≤50 nmol/L at inclusion were directly randomized. Patients with 25OHD >50 nmol/L at inclusion underwent a 3-month washout renewable three times (max. washout of 12 months) and were randomized as soon as 25OHD was ≤50 nmol/L. PTH, calcium and phosphate were measured every 3 months during the whole study.

During washout, all vitamin D supplementation (native or active) was suspended. Patients with 25OHD still >50 nmol/L after 12 months of washout were excluded from the study.

Randomized patients were randomly allocated to each arm (cholecalciferol or no treatment) in a 1:1 ratio between the two arms using a computerized random program [MINIM^©^ V1.15 (Minimisation program for allocating patients to treatments in clinical trials, Evans S, Day S, Royston P, Department of Clinical Epidemiology, The London Medical Hospital College, UK)] stratified per center, age (<70 or ≥70 years old) and gender.

During the treatment period, patients randomized to the cholecalciferol group received 100 000 IU oral cholecalciferol (UVEDOSE^®^ 100 000 IU, CRINEX, France) every month for 6 months, but no other vitamin D supplementation. Cholecalciferol was provided to the participating centers by the study coordinator and given to patients during dialysis sessions under nurse supervision. Patients randomized to the control group had no vitamin D at all. None of the patients received active vitamin D or cinacalcet during the treatment phase. All other medical treatments and dialysis prescriptions were maintained as usual care.

Exclusion criteria were: hypercalcemia >2.7 mmol/L; hyperparathyroidism as defined by the KDIGO, hypoparathyroidism (iPTH <3× normal laboratory lower value), cholecalciferol intolerance or allergy, renal transplantation or hemodialysis withdrawal, pregnancy and the inability to perform handgrip.

### Objectives and judgement criteria

The main objective of the study was to analyze whether a 6-month period of oral cholecalciferol improved the muscle strength of hemodialysis patients with low 25OHD vitamin D levels. Muscle strength was compared at randomization and at 6 months using HGS measurement with a quantitative dynamometer (Kern, Germany). Measures were performed on both arms before the hemodialysis session in a standardized procedure at randomization, and at 3 months and 6 months.

Secondary objectives were to analyze whether cholecalciferol supplementation improved patients’ autonomy and reduced frailty, and its impact on phosphate, calcium and PTH concentrations. Patient autonomy was measured by the Activities of Daily Living-Katz index (ADL) which is a standardized measure of biological and psychosocial function [29]. It is a standardized scale, developed to assess the level of functional autonomy in basic activities necessary for daily living. A score of 0 means a fully dependent patient, and the maximal score of 6 a fully independent one.

Frailty risk was measured by the "Frail Non-Disabled" auto-questionnaire (FiND) which is a validated scoring method of frailty risk [30]. In this questionnaire, a score of 0 means no frailty, a score of 1–2 pre-frailty and a score ≥3 indicates a frail state.

### Statistics

Calculation of the sample size in order to meet our main objective was based on an observational study showing a dose-dependent correlation between 25OHD and muscle strength measured by handgrip [13]. As previously published, to show a 5-kg difference between the two groups, considering a standard deviation of 8 kg, an α-risk of 0.05 and a power of 0.9, the number of patients required was 54 patients in each group.

All variables underwent descriptive analysis. Qualitative (categorical) data were described using counts and percentages, while quantitative data were presented as means and standard deviations. The chi-square (χ²) test was used to compare categorical variables between the two randomized groups, and Student’s *t*-test was applied to compare quantitative variables. When the assumptions required for these parametric tests were not checked, appropriate non-parametric tests were used, such as the Mann–Whitney U test for continuous data.

The status of patients throughout the study was described using a flow diagram, detailing the number of patients at each stage and the reasons for exclusion (e.g. included but not randomized, randomized but not treated, not evaluated or not analyzed).

The primary analysis was conducted including all randomized patients, who were analyzed according to their assigned group, regardless of the treatment received. An analysis of missing data was performed to determine the appropriate strategy for handling incomplete observations. Sensitivity analyses were conducted on different populations, including the evaluable population, the randomized population with data imputation strategies and the per-protocol population.

Secondary endpoints were analyzed using the same statistical methods as for the primary endpoint. Baseline values were defined as the last measurements taken prior to randomization. Statistical significance was set at a two-sided *P*-value <.05.

All statistical analyses were performed using Python (version 3.12.7), with the pandas and scipy libraries.

### Ethics

According to French law, this study has received approval from an ethics committee (Comité de Protection des Personnes EST III, authorization no. 17.09.07 of 06/09/2017) and the French Agency for Safety of Health Products (ANSM) has been informed.

## RESULTS

### Study population

**Figure 1:**
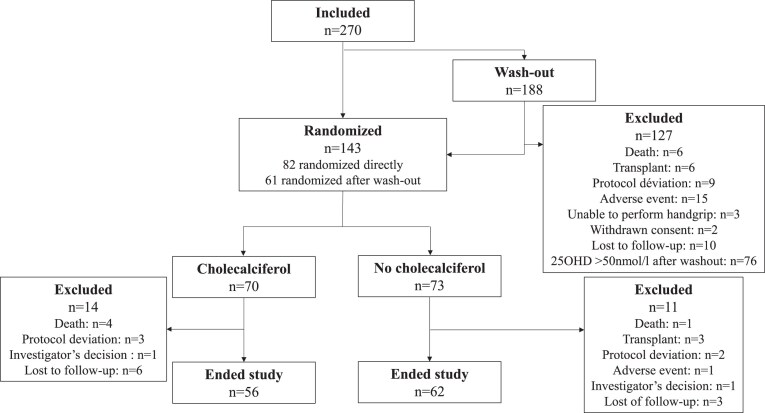
Flowchart of the VITADIAL trial. Among 270 hemodialysis patients screened, 188 entered a washout period to achieve serum 25OHD levels ≤50 nmol/L. Ultimately, 143 patients were randomized: 70 were assigned in the cholecalciferol group and 73 in the no cholecalciferol group (control group), within which 56 and 62 patients, respectively, ended the study, reaching the number of patients needed for the study.

Between January 2018 and October 2023, within the 270 hemodialysis patients from 10 hemodialysis centers who were included in the study, 82 had an 25OHD ≤50 nmol/L at inclusion and were directly randomized and the 188 with 25OHD >50 nmol/L underwent a 3–12 month wash out with no vitamin D (Fig. 1, Supplementary data, Table S1 and Fig. S1). From these 188 patients, 127 were excluded during washout and 61 reached a 25OHD ≤50 nmol/L and were randomized (Fig. 1). During the washout phase, 6 patients died, 6 received a kidney transplant, 9 were excluded due to protocol deviations (resumption of vitamin D supplementation) and 15 experienced adverse events (including 5 cases of hyperparathyroidism, 7 of hypocalcemia, 1 hip fracture, 1 vertebral fracture and 1 new diagnosis of severe osteoporosis), 2 withdrew consent and 10 were lost to follow-up. In addition, 76 patients were excluded because their 25OHD levels remained >50 nmol/L despite up to 12 months of correctly followed washout. The trial ended when the target number of patients were enrolled and completed follow-up.

At inclusion, mean of the maximal HGS was 24.8 ± 9.5 kg and mean 25OHD before washout and randomization procedures was 81.75 ± 48.5 nmol/L, with 63.5% of patients treated with vitamin D supplements at inclusion.

Maximal HGS at inclusion was higher in men than women (28.1 ± 8.12 kg versus 18.5 ± 8.85 kg, *P *< .001), and negatively correlated with age (rho=–0.23, *P *< .001), but was not influenced by diabetes (25.5 ± 9.93 in patients with diabetes, 24.1 ± 9.12 kg without; *P *= .25) or vascular access (analysis of variance *P *= .08), nor did it correlated to 25OHD (rho = 0.06, *P *= .31), body mass index (rho = 0.060, *P *= .33) or dialysis vintage (rho= –0.043, *P *= .48).

Characteristics of randomized patients did not differ from the whole included population (Table S1). The 143 patients were randomized as follows: 70 were allocated to the cholecalciferol group and 73 to the control group. Characteristics of patients at randomization are provided in Table 1. There was no difference between the two groups at randomization.

**Table 1:** Data at randomization.

	Cholecalciferol (*n* = 70)	No cholecalciferol (*n* = 73)	*P*-value
Male (%)	67.1	65.8	1.0
Age (years)	69.3 ± 11.5	69.4 ± 16.7	.434
Diabetes (%)	58.6	57.5	1.0
Dialysis vintage (year)	3.4 ± 5.3	3.1 ± 5.7	.246
BMI (kg/m²)	26.5 ± 6.3	26.9 ± 5.0	.263
Living environment (%)			.674
Home	95.7	97.3	
Institution	4.3	2.7	
Nephropathy (%)			.506
Nephrosclerosis	21.4	19.2	
Diabetes	28.6	32.9	
ADPKD	7.1	5.5	
Glomerular	12.9	9.6	
Interstitial nephritis	8.6	11	
Other	4.3	12.3	
Unknown	17.1	9.6	
Dominant hand (%)			.124
Right hand	88.6	95.9	
Left hand	11.4	4.1	
Handgrip strength (kg)			
Maximum	24.5 ± 8.8	24.5 ± 10.4	.888
Right hand	23.3 ± 9.3	23.7 ± 10.1	.948
Left hand	21.8 ± 8.5	21.0 ± 10.6	.481
Dominant hand	23.5 ± 9.1	23.7 ± 10.1	.886
Vascular access (%)			.223
Arteriovenous fistulae	72.7	71.8	
Prothesis	3.0	9.9	
Catheter	24.2	18.3	
Randomized (%)			.86
Directly	48.5	51.52	
After washout	51.5	48.48	
Charlson comorbidity score	7.15 ± 2.12	7.84 ± 2.03	.087
Dialysis parameters (%)			.620
Hemodiafiltration	40.0	39.2	
Hemodialysis	57.1	58.1	
Hemofiltration	1.4	1.4	
Other	1.4	1.4	
Number of sessions per week	3.04 ± 0.20	3.00 ± 0.16	.172
Dialysis time per session (h)	3.82 ± 0.45	3.93 ± 0.33	.152
Nutritional supplementation (%)			
Oral nutritional supplement	17.1	17.8	1.0
Intradialytic parenteral nutrition	1.4	2.7	1.0
Biological parameters			
Hemoglobin (g/dL)	11.20 ± 1.08	11.41 ± 1.18	.285
Predialysis creatinine (µmol/L)	749.12 ± 1108.75	707.48 ± 661.62	.836
Albumin (g/L)	37.43 ± 3.63	36.83 ± 4.30	.387

Data are presented as mean ± standard deviation or *n* (%). ADPKD, autosomal dominant polycystic disease; BMI, body mass index. *P*-values are provided for comparison between the cholecalciferol and no cholecalciferol groups.

During the study, 14 patients of the cholecalciferol group and 11 patients from the control group were excluded. Thus, 56 and 62 patients, respectively, were analyzed at the end of the study, reaching the target sample size required for the study (Fig. 1).

### 25OHD concentrations

**Figure 2:**
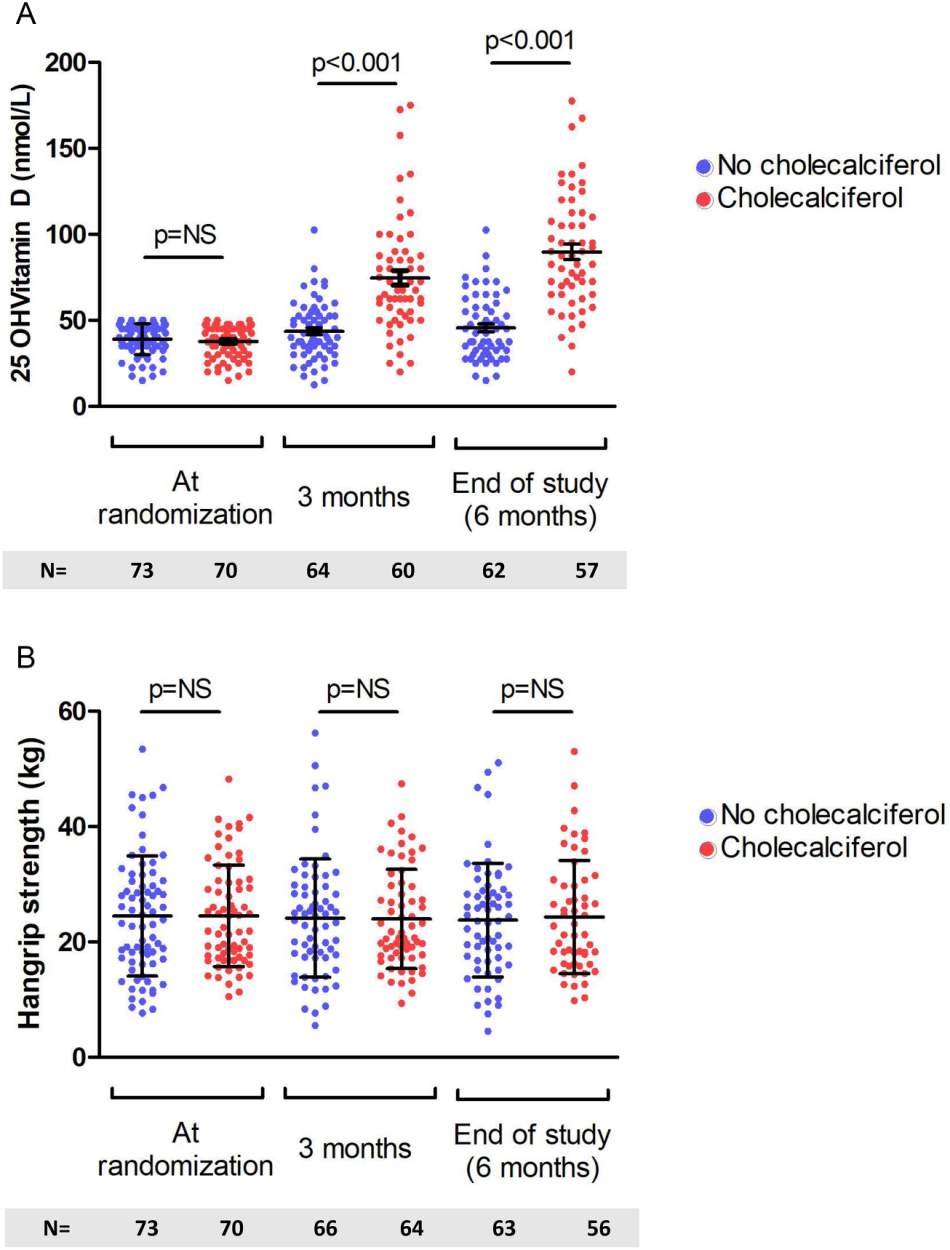
25OH D concentrations and HGS values. (**A**) 25OHD concentrations. Each dot represents a patient. (**B**) Maximal HGS between both arms. Each dot represents a patient. The number of patients may differ between panels because each graph includes all available measurements for the corresponding variable at each time point.

At randomization, mean 25OHD were comparable in the cholecalciferol and control group (respectively 37.75 ± 9.43 and 39.0 ± 9.05 nmol/L; *P *= .404) (Table 2, Fig. 2A). In the cholecalciferol-supplemented group, 25OHD increased to 74.63 ± 33 nmol/L at 3 months and 89.78 ± 33.43 nmol/L at 6 months, which was significantly different from baseline (*P *< .001 for the two values). In the no cholecalciferol group, 25OHD at 3 and 6 months were, respectively, 43.63 ± 16.88 and 45.56 ± 18.35 nmol/L, remaining <50 nmol/L for most patients. The 25OHD concentrations at 3 months and at 6 months were significantly different in the two groups (*P *< .001).

**Table 2:** Evolution of secondary objective parameters during the study.

		Randomization	At 3 months	At end of study (6 months)	Change between randomization and end of study
25OHD (nmol/L)	Cholecalciferol	37.75 ± 9.43	74.63 ± 33.0***	89.78 ± 33.43^§§§^	51.7 ± 33.8
	No cholecalciferol	39.08 ± 9.05	43.63 ± 16.88	45.56 ± 18.35^§^	6.13 ± 17.85
	*P*-value	.404	<.001	<.001	<.001
Calcium (mmol/L)	Cholecalciferol	2.14 ± 0.17	2.16 ± 0.19	2.19 ± 0.19^§^	0.06 ± 0.16
	No cholecalciferol	2.23 ± 0.45	2.28 ± 0.82	2.17 ± 0.16	–0.07 ± 0.48
	*P*-value	.069	.594	.520	.009
Phosphate (mmol/L)	Cholecalciferol	1.61 ± 0.69	1.53 ± 0.55	1.52 ± 0.55	–0.11 ± 0.57
	No cholecalciferol	1.84 ± 0.91	1.73 ± 0.68	1.52 ± 0.47	–0.30 ± 0.92
	*P*-value	.085	.120	.840	.268
PTH (ng/L)	Cholecalciferol	309.57 ± 237.64	260.25 ± 164.45*	225.36 ± 155.29^§§^	–99.06 ± 243.20
	No cholecalciferol	265.44 ± 193.10	243.86 ± 160.56	256.03 ± 200.63	–8.53 ± 207.41
	*P*-value	.159	.437	.606	.021
ADL	Cholecalciferol	5.61 ± 0.84	No data	5.50 ± 1.19	–0.05 ± 1.03
	No cholecalciferol	5.38 ± 1.14	No data	5.40 ± 1.17	0.09 ± 0.61
	*P*-value	.420		.427	.565
FiND	Cholecalciferol	2.19 ± 1.56	No data	2.14 ± 1.55	–0.08 ± 1.42
	No cholecalciferol	2.22 ± 1.65	No data	2.28 ± 1.62	–0.02 ± 1.18
	*P*-value	.924		.684	.827

*Represents a significant difference between randomization and 3 months (*P < .05; **P < .01; ***P < .001). ^§^Represents a significant difference between randomization and end of study (^§^*P* < .05; ^§§^*P* < .01; ^§§§^*P* < .001). *P*-values are provided for the comparison between cholecalciferol and no cholecalciferol groups.

### Handgrip strengths

At randomization, HGS in the cholecalciferol and no cholecalciferol groups were not statistically different (respectively, 24.54 ± 8.79 and 24.49 ± 10.41; *P *= .888) (Table 3, Fig. 2B). HGS did not change significantly between randomization and end of the study in each group (0.07 ± 3.40 kg and –0.19 ± 3.35 kg, respectively; *P *= .682). Cholecalciferol had no effect on HGS during the study, even when men and women were analyzed separately (Table 3, Supplementary data, Fig. S2A and B), or when we studied the highest value between both arms or when we studied only the dominant arm (data not shown). We then analyzed the subgroup of cholecalciferol supplemented patients for whom 25OHD increased over the normal value of 75 nmol/L and also found no effect of cholecalciferol on HGS (data not shown). Identically, there was no effect of cholecalciferol on HGS in the subgroup of patients with 25OHD <15 nmol/L at randomization (data not shown).

**Table 3:** HGS measurements in randomized patients.

		Randomization	3 months	End of study (6 months)	Change in HGS
Total population	Cholecalciferol	24.54 ± 8.79	23.96 ± 8.59	24.31 ± 9.81	0.07 ± 3.40
	No cholecalciferol	24.49 ± 10.41	24.12 ± 10.25	23.62 ± 9.85	–0.19 ± 3.35
	*P*-value	.888	.987	.923	.682
Men	Cholecalciferol	27.51 ± 8.38	27.21 ± 7.97	28.49 ± 9.72	0.22 ± 3.76
	No cholecalciferol	28.61 ± 8.94	28.07 ± 8.29	27.25 ± 8.44	–0.75 ± 3.44
	*P*-value	.536	.715	.659	.259
Women	Cholecalciferol	18.46 ± 6.18	18.16 ± 6.40	18.32 ± 6.27	–0.14 ± 2.86
	No cholecalciferol	16.57 ± 8.34	17.17 ± 10.17	17.47 ± 9.11	0.77 ± 3.02
	*P*-value	.063	.231	.429	.298

Data are provided in kilograms for the highest value between the two arms (max. handgrip of the patient). Change in HGS are provided in comparison with HGS at randomization. *P*-values are provided for the comparison between cholecalciferol and no cholecalciferol groups. There was no significant change between the HGS values at randomization and at 3 months, or between values at randomization and at end of study in any group.

During the washout period, no significant change in HGS was observed in the study population (Supplementary data, Fig. S3).

### CKD mineral and bone disorder parameters

At randomization, calcium concentrations were similar in the two groups (respectively, 2.14 ± 0.17 mmol/L in the intervention group and 2.23 ± 0.45 in the control group; *P *= .069) (Table 2). During the study, calcium concentration increased to 2.19 ± 0.19 mmol/L in the cholecalciferol supplemented patients (*P *< .05), but did not change significantly in the no cholecalciferol group.

At randomization, phosphate concentrations were similar in the two groups (respectively, 1.61 ± 0.69 mmol/L in the intervention group and 1.84 ± 0.91 in the control group; *P *= .085) (Table 2). There was no change in phosphate concentrations of each study group during the study.

At randomization, PTH concentrations were comparable between the two groups (respectively, 309.57 ± 237.64 ng/L in the intervention group vs 265.44 ± 193.10 ng/L in the control group; *P *= .159) (Table 2). PTH concentrations significantly decreased in the cholecalciferol group but did not change in the control group (*P *= .02).

### ADL and FiND

At baseline, the mean ADL score was comparable between the cholecalciferol and control groups (5.61 ± 0.84 vs 5.38 ± 1.14; *P *= .42), indicating a generally high level of autonomy in both cohorts. After 6 months of follow-up, no significant difference was observed between groups. The mean change in ADL score was –0.05 ± 1.03 in the vitamin D group versus 0.09 ± 0.61 in the control group (*P *= .57) (Table 2).

At baseline, the mean FiND score was comparable between the cholecalciferol and control groups (2.19 ± 1.56 vs 2.22 ± 1.65; *P *= .924). Within all randomized patients, 19.3% were not frail (score of 0), 49.3% had a pre-frail state (score 1–2) and 41.5% were frail (score 3–5). During the study, the mean FiND score did not change significantly in the two groups (Table 2).

### Adverse events

During the washout period, 15 patients experienced adverse events and were excluded from the study: 5 patients developed hyperparathyroidism, 7 had episodes of hypocalcemia, 1 sustained a hip fracture, another a vertebral fracture and 1 patient was diagnosed with severe osteoporosis necessitating vitamin D supplementation.

During the treatment phase, 14 patients from the cholecalciferol group and 11 from the control group (no cholecalciferol) were excluded for reasons detailed in Fig. 1. Notably, the only adverse event reported during this phase was a case of hypocalcemia occurring in a patient assigned to the control group.

## DISCUSSION

In this multicentric randomized controlled study, specifically designed and powered to assess whether cholecalciferol supplementation in hemodialysis patients with low 25OHD improves muscle strength, we found that despite a significant increase in serum 25OHD concentrations, cholecalciferol supplementation did not improve HGS after 6 months. To our knowledge, this is the first study in the field powered enough to conclude on the lack of effect of cholecalciferol on muscle strength in this population: the target number of patients was reached, groups were comparable at inclusion and patients randomized had low 25OHD at inclusion, and with monthly oral 100 000 IU cholecalciferol supplementation, their mean 25OHD reached the expected threshold of >75 nmol/L [31]. Our results do not support the use of vitamin D supplementation with the expectation of improving muscle strength in hemodialysis patients.

Although cholecalciferol was administrated orally during the dialysis session under nurse oversight, all patients in the cholecalciferol group did not reach the normal 25OHD level of 75 nmol/L. Nevertheless, even in the subgroup of patients reaching the threshold of 75 nmol/L, there was no effect of vitamin D supplementation on muscle strength.

Cholecalciferol supplementation had no significant impact on the autonomy assessed by the patient with the ADL or frailty assessed by the medical team (FiND) over the 6-month study period in hemodialysis patients with baseline 25OHD levels ≤50 nmol/L.

Our results are in accordance with previous studies in the field that were less powered, but already reported no effect of cholecalciferol on muscle strength in hemodialysis patients [25–27]. In the general population, cholecalciferol’s effect on muscle strength and falls remains controversial: large meta-analysis might suggest a small effect in elderly with very low 25OHD [20–22], but this effect seems very limited.

Interestingly, the population enrolled in this clinical trial displayed similar characteristics than the overall French hemodialysis population, as reported in the Renal Epidemiology and Information Network (REIN) registry, which encompasses all hemodialysis patients in France. In this registry, in 2023, the median age of prevalent hemodialysis patients was 71.3 years, with a sex ratio of 1.8 and a median dialysis vintage of 3.4 years (www.agence-biomedecine.fr). Therefore, our findings may be reasonably extrapolated to the broader French and European hemodialysis population.

Numerous observational studies have reported a positive association between 25OHD and muscle strength or physical performance, in the general population [14–16] and in hemodialysis patients, but most interventional studies remain negative [13, 32]. Vitamin D deficiency is highly prevalent in populations with poor nutritional status, chronic inflammation, reduced physical activities and chronic conditions such as kidney failure, all of which independently contribute to sarcopenia [8, 9]. Thus, low 25OHD concentrations may reflect a global state of bad health, frailty or protein-energy wasting rather than directly causing impaired muscle function: cholecalciferol is mainly a good integrative biomarker.

As already discussed, muscle dysfunction in hemodialysis patients results from many factors: protein-energy wasting syndrome, chronic inflammation, metabolic acidosis, low physical activity, uremic toxins such as indoxyl sulfate or myostatin accumulation [5–9]. Thus, a unique intervention such as cholecalciferol supplementation might be inefficient on its own, and a multimodal approach to strive against sarcopenia including optimized nutritional support, physical activity program and potentially pharmacological approaches targeting pathways may hold greater promise.

In our study, cholecalciferol had a statistically significant effect on calcium concentrations, but the increase was small and the clinical relevance of this effect remains to be determined. Cholecalciferol also efficiently lowered PTH, and had no effect on phosphate concentrations. These effects are those usually obtained with native vitamin D treatments in 25OHD-deficient hemodialysis patients [33].

Our study also has limitations: for feasibility reasons, our study is open labelled, but our main objective is based on an objective measure of muscle strength which limits the bias. Could a 6-month follow-up be too short to see an effect of cholecalciferol supplementation? Probably not, as we found no tendency at 6 months and others did not find any effect at 12 months. Finally, we only measured muscle strength, and did not explore endurance or muscle mass in our study, but our study did not find any functional effect on daily activities.

The inclusion threshold of 25OHD ≤50 nmol/L may have selected patients with low vitamin D status but not always profound deficiency according to other guideline definitions. Although the subgroup analyses of patients with 25OHD <15 nmol/L at randomization did not show any signal of benefit, still, this may have reduced the ability to detect a benefit of supplementation on muscle strength, and patients with more severe deficiency might require a longer duration of treatment or a different dosing strategy.

Of note, our results with cholecalciferol should not be extrapolated to other vitamin D compounds, but to date, no other vitamin D compound has proved any effect on muscle strength or mass in this population.

A monthly 100 000 IU cholecalciferol regimen was used in this trial which was designed in 2017. High doses of cholecalciferol have been associated with an increase in falls and fractures, and recent European consensus guidance recommends avoiding mega-doses (≥100 000 IU) in CKD [34]. Thus, more frequent and lower dosing of supplementation should now be preferred.

Serum 1,25(OH)2D was not measured in this study, and therefore we could not explore the heterogeneity of vitamin D activation in dialysis patients, including in patients with markedly impaired or absent renal 1α-hydroxylation such as nephrectomized patients. This should be considered when interpreting the biological effects of cholecalciferol in this population.

In conclusion, this well-powered randomized controlled study did not find any effect of cholecalciferol supplementation on muscle strength of hemodialysis patients with low 25OHD. Cholecalciferol could be prescribed to increase calcium concentrations and lower PTH, but not to increase muscle strength.

## Supplementary Material

sfag166_Supplemental_Files

## Data Availability

Anonymized data that support the findings of this study are available from the corresponding author upon reasonable request and subject to ethical approval.
