# Supplementary material for: Cholecalciferol and muscle strength in hemodialysis patients: results from the randomized VITADIAL trial
Source: Clin Kidney J. 2026 May 21;19(7):sfag166. doi: 10.1093/ckj/sfag166 (PMC13320236; doi:10.1093/ckj/sfag166)
Supplement: sfag166_Supplemental_Files [file sfag166_supplemental_files.zip › 1905 Supplementary table 1.docx]

**Supplementary table 1:** **Data at inclusion compared between the whole cohort of included patients and the patients randomized.** ADPKD: autosomal dominant polycystic kidney disease; BMI: body mass index. P-values are provided for comparison between Included and Randomized patients.

|  | **Included**  **n=270** | **Non randomized**  **n=127** | **Randomized**  **n=143** | **p-value** |
| --- | --- | --- | --- | --- |
| Male | 64.1 % | 61.4% | 66.4% | 0.712 |
| Age (years) | 70.8 ± 13.4 | 72.4 ± 12.1 | 69.3±14.4 | 0.419 |
| Diabetes | 52.6 % | 46.5% | 58.04% | 0.340 |
| Dialysis vintage (year) | 3.39 ± 5.5 | 3.56 ± 5.4 | 3.24 ± 5.49 | 0.271 |
| BMI (kg/m²) | 26.9 ± 6.0 | 27.2 ± 6.6 | 26.6 ± 5.53 | 0.762 |
| Living environment  Home  Institution  other | 97.0%  2.6%  0.4% | 97.6%  1.6%  0.8% | 95.8%  3.5%  0% | 0.996 |
| Nephropathy  Nephrosclerosis  Diabetes  ADPKD  Glomerular  Interstitial nephritis  Other  Unknown | 20.7%  25.9%  7.8%  12.2%  10.0%  8.5%  14.8% | 21.3%  20.5%  9.4%  13.4%  10.2%  8.7%  16.5% | 20.3%  30.8%  6.3%  11.2%  9.8%  8.4%  13.3% | 0.970 |
| Dominant hand  Right hand  Left hand | 93.2%  6.8% | 94.3%  5.7 | 92.3%  7.7 | 0.829 |
| Handgrip strength (kg)  Maximum  Right hand  Left hand  Dominant hand | 24.8 ± 9.5  23.8 ± 9.1  21.9 ± 9.6  23.8 ± 9.2 | 25.0 ±8.8  24.4 ±9.1  21.6 ± 8.7  24.4 ± 9.0 | 24.6 ± 10.1  23.4 ± 9.6  21.6 ± 10.4  23.8 ± 9.3 | 0.899  0.518  0.516  0.501 |
| Vascular access  AV fistulae  Prothesis  Catheter | 75.1%  7.8%  17.1% | 78.3  9.2  12.5 | 72.3  6.6  21.1 | 0.587 |
