# Supplementary material for: Cholecalciferol and muscle strength in hemodialysis patients: results from the randomized VITADIAL trial
Source: Clin Kidney J. 2026 May 21;19(7):sfag166. doi: 10.1093/ckj/sfag166 (PMC13320236; doi:10.1093/ckj/sfag166)
Supplement: sfag166_Supplemental_Files [file sfag166_supplemental_files.zip › Supp Fig 1 Nombre de patients par centre V2.pptx]

## Slide 1
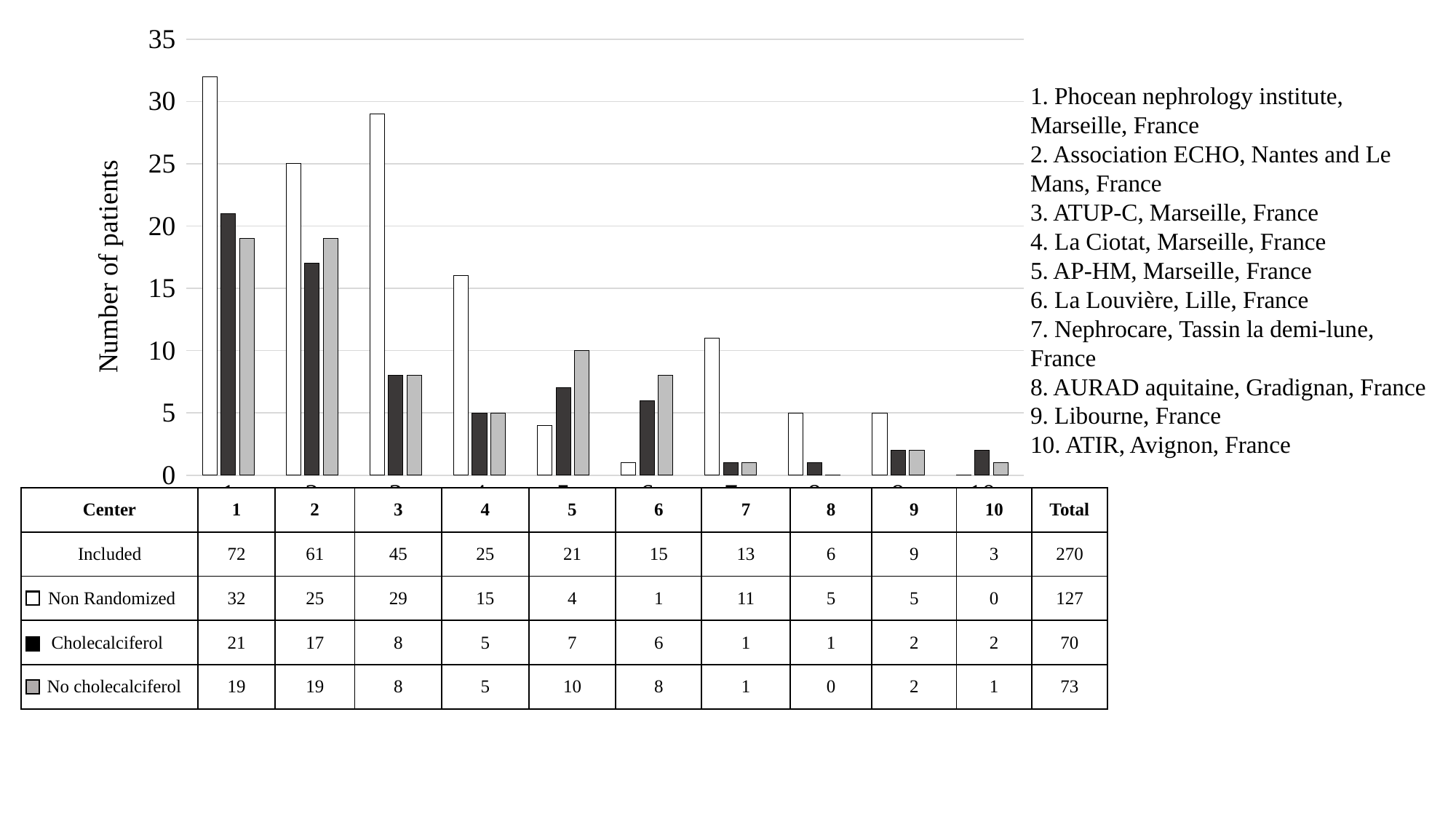

### Chart
| Category | Non randomized | Cholecalciferol | No cholecalciferol |
|---|---|---|---|
| 1 | 32.0 | 21.0 | 19.0 |
| 2 | 25.0 | 17.0 | 19.0 |
| 3 | 29.0 | 8.0 | 8.0 |
| 4 | 16.0 | 5.0 | 5.0 |
| 5 | 4.0 | 7.0 | 10.0 |
| 6 | 1.0 | 6.0 | 8.0 |
| 7 | 11.0 | 1.0 | 1.0 |
| 8 | 5.0 | 1.0 | 0.0 |
| 9 | 5.0 | 2.0 | 2.0 |
| 10 | 0.0 | 2.0 | 1.0 |1. Phocean nephrology institute, Marseille, France
2. Association ECHO, Nantes and Le Mans, France
3. ATUP-C, Marseille, France
4. La Ciotat, Marseille, France
5. AP-HM, Marseille, France
6. La Louvière, Lille, France
7. Nephrocare, Tassin la demi-lune, France
8. AURAD aquitaine, Gradignan, France
9. Libourne, France
10. ATIR, Avignon, France
| Center | 1 | 2 | 3 | 4 | 5 | 6 | 7 | 8 | 9 | 10 | Total |
| --- | --- | --- | --- | --- | --- | --- | --- | --- | --- | --- | --- |
| Included | 72 | 61 | 45 | 25 | 21 | 15 | 13 | 6 | 9 | 3 | 270 |
| Non Randomized | 32 | 25 | 29 | 15 | 4 | 1 | 11 | 5 | 5 | 0 | 127 |
| Cholecalciferol | 21 | 17 | 8 | 5 | 7 | 6 | 1 | 1 | 2 | 2 | 70 |
| No cholecalciferol | 19 | 19 | 8 | 5 | 10 | 8 | 1 | 0 | 2 | 1 | 73 |
