# Supplementary figures and images for: Cholecalciferol and muscle strength in hemodialysis patients: results from the randomized VITADIAL trial
Source: Clin Kidney J. 2026 May 21;19(7):sfag166. doi: 10.1093/ckj/sfag166 (PMC13320236; doi:10.1093/ckj/sfag166)

## Slide 1
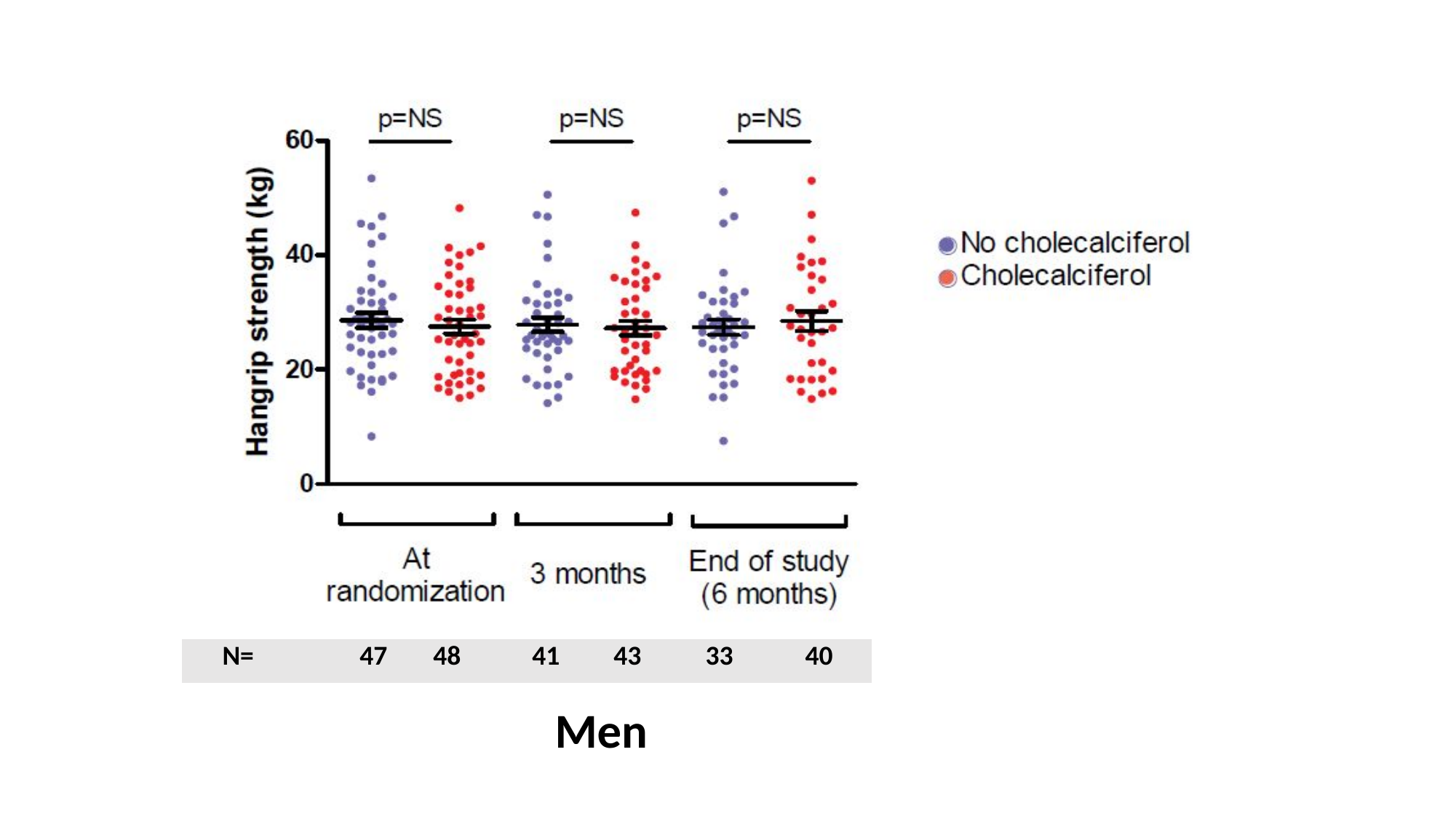

| N= | 47 | 48 | 41 | 43 | 33 | 40 |
| --- | --- | --- | --- | --- | --- | --- |
Men

## Slide 2
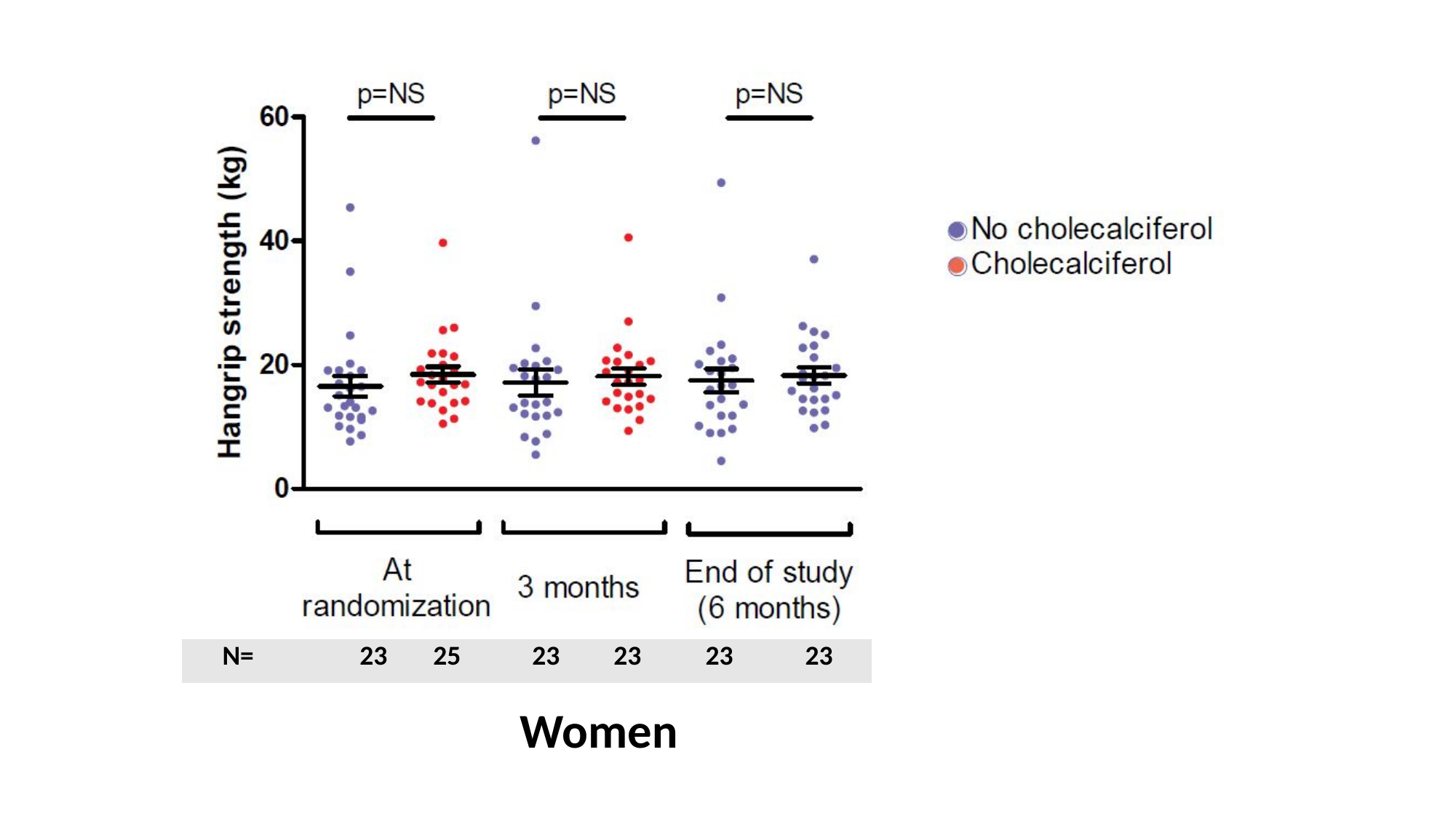

| N= | 23 | 25 | 23 | 23 | 23 | 23 |
| --- | --- | --- | --- | --- | --- | --- |
Women

Supplement: sfag166_Supplemental_Files [file sfag166_supplemental_files.zip › Supp Fig 2A et B Par sexe.pptx]

## Slide 1
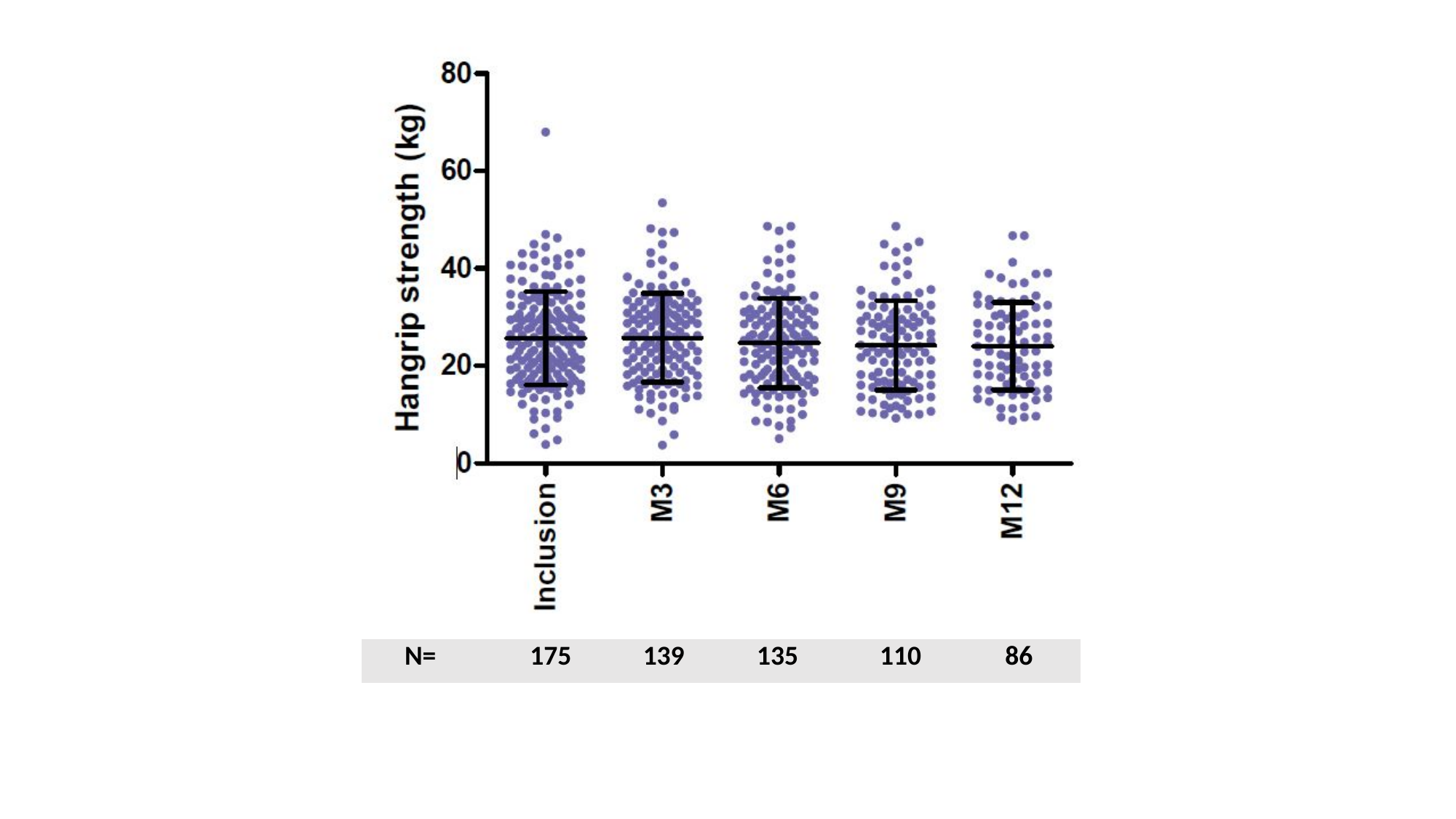

| N= | 175 | 139 | 135 | 110 | 86 |
| --- | --- | --- | --- | --- | --- |

Supplement: sfag166_Supplemental_Files [file sfag166_supplemental_files.zip › Supp Fig 3.pptx]
